# Supplementary material for: Liposome-based transfection enhances RNAi and CRISPR-mediated mutagenesis in non-model nematode systems
Source: Sci Rep. 2019 Jan 24;9:483. doi: 10.1038/s41598-018-37036-1 (PMC6345965; doi:10.1038/s41598-018-37036-1)
Supplement: Supplementary file 1 — Supplementary Information [file 41598_2018_37036_MOESM1_ESM.docx]

**Title: Liposome-based transfection enhances RNAi and CRISPR mediated mutagenesis in non-model nematode systems.**

**Authors: Sally Adams^1#^, Prachi Pathak^1#^ Hongguang Shao^2^, James B. Lok^2^ and Andre Pires-daSilva^1^***

**Supplemental Table 1: List of primers used**

| Name | Species | DNA sequence (5’ to 3’)  (T7 promoter sequence underlined if present) | Application |
| --- | --- | --- | --- |
| UW500  UW501 | *A. rhodensis* | TAATACGACTCACTATAGGGGCCCGACAAATTCAAGAACGCCG  TAATACGACTCACTATAGGGAGACATCCACCTCCGGTCCATCA | *Arh-par-1* dsRNA synthesis |
| UW219  UW220 | *A. freiburgensis* | TAATACGACTCACTATAGGGTCTAAGCCTACAAGCCAGGAGCC  TAATACGACTCACTATAGGGAAAAGCTCTGGTGCGGCATATGG | *Afr-par-1* dsRNA synthesis |
| UW510  UW511 | *P. pacificus* | **TAATACGACTCACTATAGGG**TAATGAAGCAGCTGGACCACCCG  **TAATACGACTCACTATAGGG**GCAGTTCGTCAAACTTGTCCGCC | *Ppa-par-1* dsRNA synthesis |
| UW233  UW234 | *A. freiburgensis* | TAATACGACTCACTATAGGGTGGCTGTAATGGGTCCAGTTGGT  TAATACGACTCACTATAGGGACAGCCAAGCCCATGTTTGAAGG | *Afr-unc-22* dsRNA synthesis |
| UW24  UW25 | *A. rhodensis* | TAATACGACTCACTATAGGGTCCAACTGACCCACAAATGA  TAATACGACTCACTATAGGGCCCATTATCACGAGGAGCAT | *Arh-unc-22* dsRNA synthesis |
| UW174  UW175 | *A. freiburgensis* | TCTTTCTGGAGCCACTTCTGGGG  ACAGTACTCGGCATCTTTTCCTGG | *Afr-rol-6.1* genomic  DNA amplification and  genotyping |
| UW159  UW555 | *A. rhodensis* | TCGACAATGAAATGGATAACTTCAAGG  TTGTTGCTAAGAGAGCACTGGC | *Arh-rol-6.1* genotyping |

**Supplemental Table 2: List of crRNA and donor fragments used for CRISPR-Cas9 mediated *rol-6* gene editing**

| Target gene | Nucleotide sequence of crRNAs pairs (gene-specific sequence in italics)  and ssDNA donor fragment (bases to be modified upon conversion bold and underlined) used in conjunction for *rol-6* gene conversion | |
| --- | --- | --- |
| *Arh-rol-6.1* | crRNA-1 (UW271)    crRNA-2 (UW272)  donor fragment  (UW92) | GUUAGACGUCAAAACUAUGGGUUUUAGAGCUAUGCU  CAUAGUUUUGACGUCUAACAGUUUUAGAGCUAUGCU  TGGACTGATATGGTCAAGCTCGGAGCTGGATCAGCCAGTAACCGTGTT**C**G**CT**G**C**CAAAACTATGGAGGATATGGAGCCAGTGGGGTTCAGCCACCAGCAG |
| *Afr-rol-6.1* | crRNA-1 (UW274)  crRNA-2 (UW306)  donor fragment  (UW323) | GUUAGAAGACAAAACUAUGGGUUUUAGAGCUAUGCU  CAUAGUUUUGUCUUCUAACAGUUUUAGAGCUAUGCU  TGGACAGATATGGTGAAACTCGGTGCAGGAACTGCTGGAAACCGTGTT**C**G**CT**G**C**CAAAACTATGGTGGGTATGGAGCAAGTGGAGTTCAACCACCTGCAG |
| *Sst-(SSTP_0000742500)* | crRNA-F (UW327)  crRNA-R (UW328)  donor fragment (UW329) | AUUAGAUGCCAAUCAUAUGGGUUUUAGAGCUAUGCU  UGGCAUCUAAUUCUGUUAGAGUUUUAGAGCUAUGCU  GAATGATATGGTAAAACTTGGTGCTGCTTCTCCATCTAACAGAATTAGA**T**G**C**CAATCATATGGTGGTTATGCTGCTGCAGGAACTCCTCCACCAACTCCA |

A)


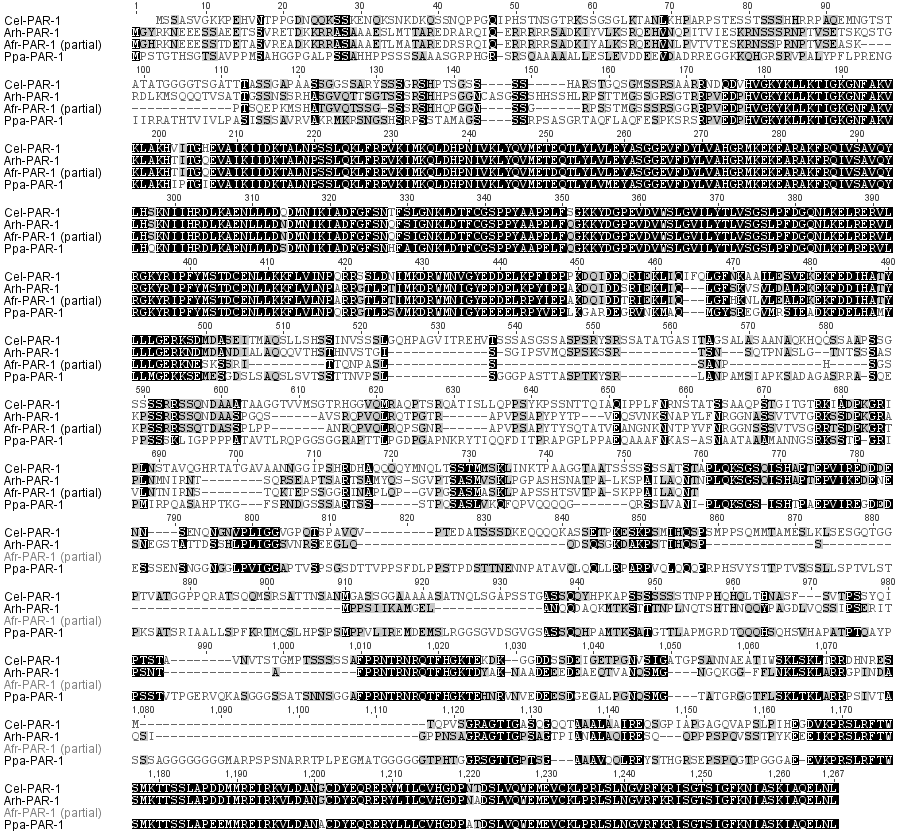


B)


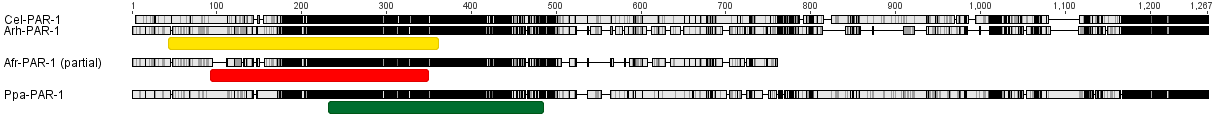


Figure S1

Sequence alignment of PAR-1 candidates. (A) The predicted proteins encoded by *Arh-par-1* (MH249768), *Afr-par-1* (MH249770) and *Ppa-par-1* (PPA12901) exhibit 49, 48 and 43% identity with the *C. elegans* PAR-1 protein (H39E23.1a) respectively. *Arh*-PAR-1 and *Afr*-PAR-1 share 75% identity with each other. A high level of conservation is observed in the predicted N- terminal kinase domain (residues 180 to 428) amongst all the proteins. (B) Regions targeted by dsRNA for RNAi are highlighted below the sequence for *Arh-par-1* (yellow), *Afr-par-1* (red) and *Ppa-par-1* (green).

A)


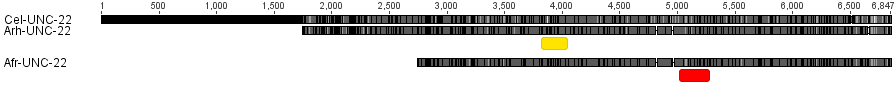


B)


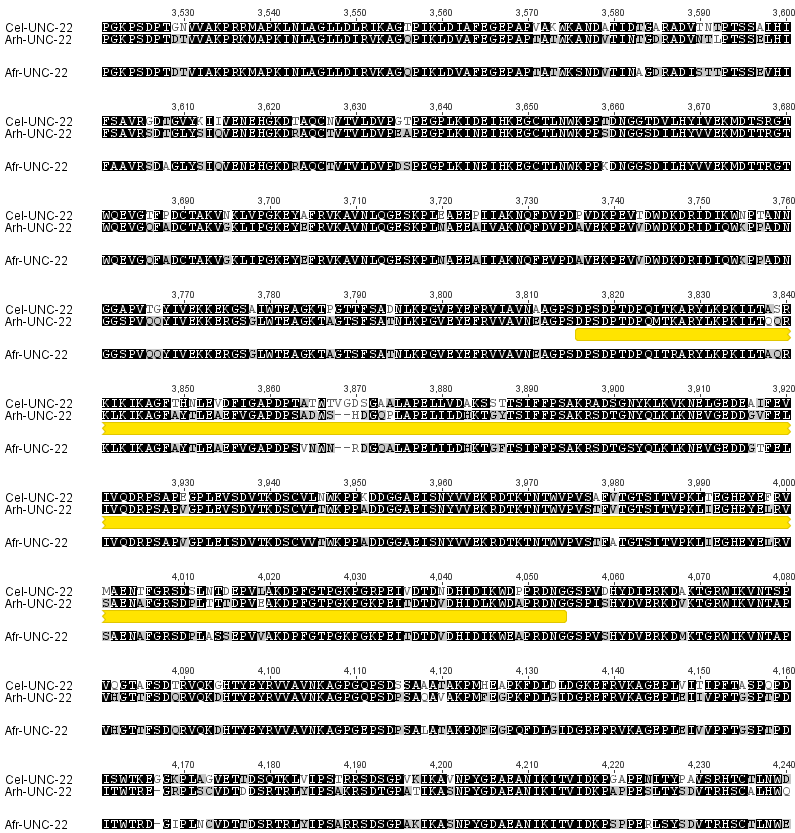


C)


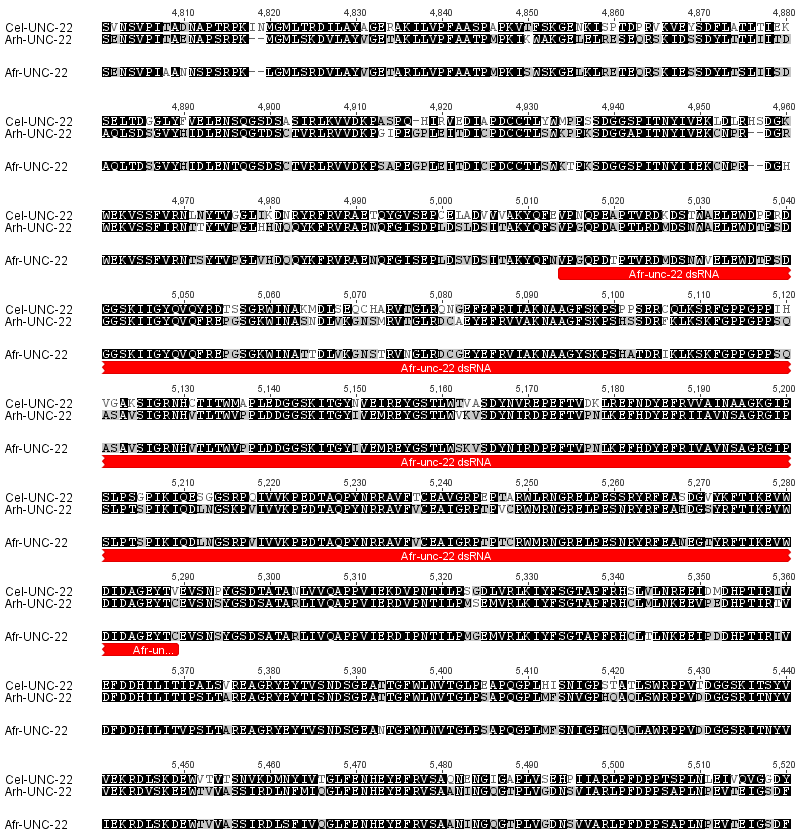


Figure S2

Sequence alignment of predicted UNC-22 proteins encoded by *Cel-unc-22*, *Arh-unc-22* (MH249769) and *Afr-unc-22* (MH124555). A) The region targeted by the dsRNA are highlighted below the sequence for *Arh-unc-22* (yellow) and *Afr-unc-22* (red). B) Alignment of UNC-22 candidate proteins in region corresponding to the *Arh-unc-22* dsRNA (shown in yellow) C) Alignment of UNC-22 candidate proteins in region corresponding to the *Afr-unc-22* dsRNA fragment (shown in red).


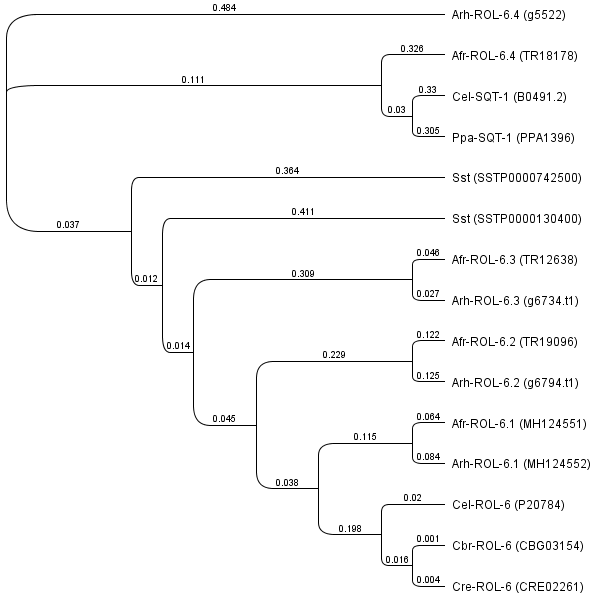


Figure S3

Tree alignment of ROL-6 and SQT-1 like predicted proteins from *A. rhodensis*, *A. frieburgensis, S. sterocoralis, P. pacificus and Caenorhabditis* species*. Arh*-ROL-6.1 and *Afr*-ROL-6.1 exhibit the highest level of homology with *Cel*-ROL-6 (62.2% and 60.9%). SSTP_0000742500 also exhibits slightly higher overall homology to *Cel*-ROL-6 than SSTP_0000130400) (45.0% compared to 44.0%). SSTP_0000742500 also exhibits a higher level of homology to *Cel*-ROL-6 compared to the related collagen protein *Cel*-SQT-1 (45.0% compared to 34%). Tree was assembled in Geneious 7.1.7. Global alignment with free end gaps using default settings (Blosum 62 matrix and Jukes Cantor model). Substitutions per site shown. Sequences for *Arh-rol-6.2* to *Arh-rol-6.4* and *Afr-rol-6.2* to *Afr-rol-6.4* available upon request.


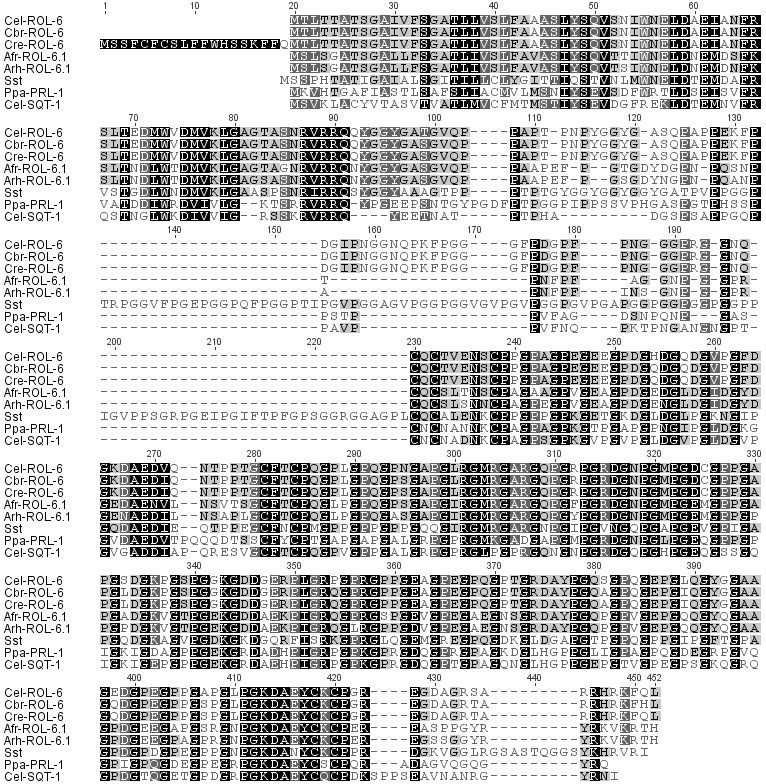


Figure S4

Alignment of full length predicted ROL-6 or SQT-1 like proteins encoded by *Cel-rol-6* (P20784), *Cbr-rol-6* (CBG03154), *Cre-rol-6* (CRE02261), *Arh-rol-6.1* (MH124552), *Afr-rol-6.1* (MH124551), *Sst* (SSTP_0000742500), *Ppa-prl-1* (which encodes for an identical protein to *Ppa-sqt-1* (PPA1396)) and *Cel-sqt-1* (B0491.2). All predicted proteins show conservation of the arginine residue (R) at position 90 above, which is converted to a cysteine in the *C. elegans* *rol-6* (*su1006*) allele or *Ppa-prl-1* (*tu92*) in *P. pacificus*, causing a dominant roller phenotype ^1,2^.

TGTCTCTTTCTGGAGCCACTTCTGGGGCCCTACTCTTCTCTGGAGCTACTTTGATCGTTTCTCTGTTTGCTGTAGCTTCTATTTATTCTCAAGTTACAACTATATGGAATGAGCTTGATAATGAAATGGACAGCTTCAAGGTAAATATCACAAATAAAACTCACAAACAAATTGCTTTTTTTTTACAGTCTTTGACCAACGACTTATGGACAGATATGGTGAAACTCGGTGCAGGAACTGCTGGAAACCGTGTTAGAAGACAAAACTATGGTGGGTATGGAGCAAGTGGAGTTCAACCACCTGCAGCTCCTGAGTTCCCTGGAACAGGAGATTATGACGGACCTAACCCACAATCCAACCCAACTCCTAATTTCCCATTCGCTGGAGGAAACCCAGGAGGACCTCGTTGTCAATGCTCTTTGACTAATAGCTGTCCTGCTGGAGCAGCTGGACCCGTTGGAGAAGCTGGACCAGATGGCGAAGATGGTCTTGATGGAATTGACGGTTATGATGGAGAGGATGCTGAGAACGTCCTCAACTCTGTCACTTCTGGTTGCTTTACTTGCCCACAAGGTCTTCCAGGACCCCAAGGACCATCTGGAGCTCCAGGAATTCGTGGAATGCGCGGAGCTAGAGGACAGCCAGGTTTCCCAGGACGTGACGGAAACCCAGGAATGCCAGGAGAAATGGGACCACCAGGAGCTCCAGGAGCTGACGGAAAAGTCGGAACTCCAGGAGAAAAAGGAGATGATGCCGAGAAACCAATTGGACGTCAAGGACCACGTGGATCTCCAGGAGAAGTTGGACCAGAAGGAGCCGAAGGAAACTCAGGTCGTGATGCCTACCCCGGACAACCAGGACCAGTTGGAGAACCAGGACAACAAGGATATCAAGGAGCTGCTGGACCTGACGGTGAAGAAGGAGCTCCAGGATCTCGTGGAAATCCAGGAAAAGATGCCGAGTACTGT

Figure S5

Fragment of *Afr-rol-6.1* genomic DNA. Currently no genome is available for *A. freiburgensis.* Therefore, a genomic region surrounding the target site for gene editing was amplified using the primers UW174 and UW175 (see methods and Table S1) and the resulting fragment sent for Sanger sequencing with the primers used for amplification. Alignment with *Afr-rol-6.1* cDNA highlighted a single intron (underlined). The PAM sites used are highlighted in yellow and the codon targeted for conversion highlighted in red.


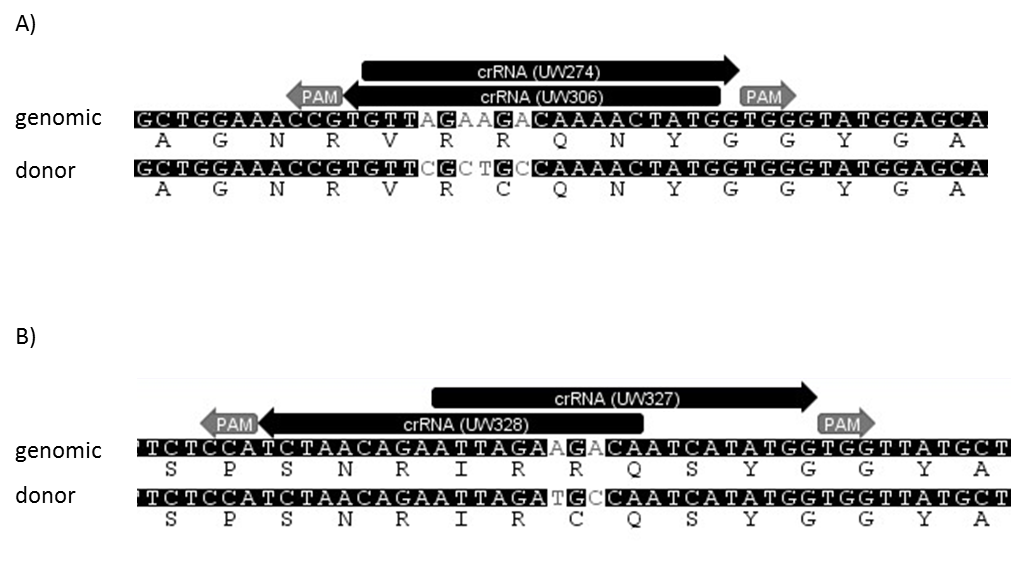


Figure S6

Sites of CRISPR-mediated gene editing in (A) *A. freiburgensis* (*Afr-rol-6.1*) and (B) *S. stercoralis* (SSTP_0000742500). Position of gene specific regions of the anti-parallel crRNA pairs are shown as black arrows and their corresponding PAM sites shown as grey arrows. crRNAs represented by forward arrows bind to the non-coding strand, whilst those shown as reverse arrows bind the coding strand. Used in combination the crRNA antiparallel pairs should result in Cas9-mediated double stranded DNA breaks either side of the targeted region.


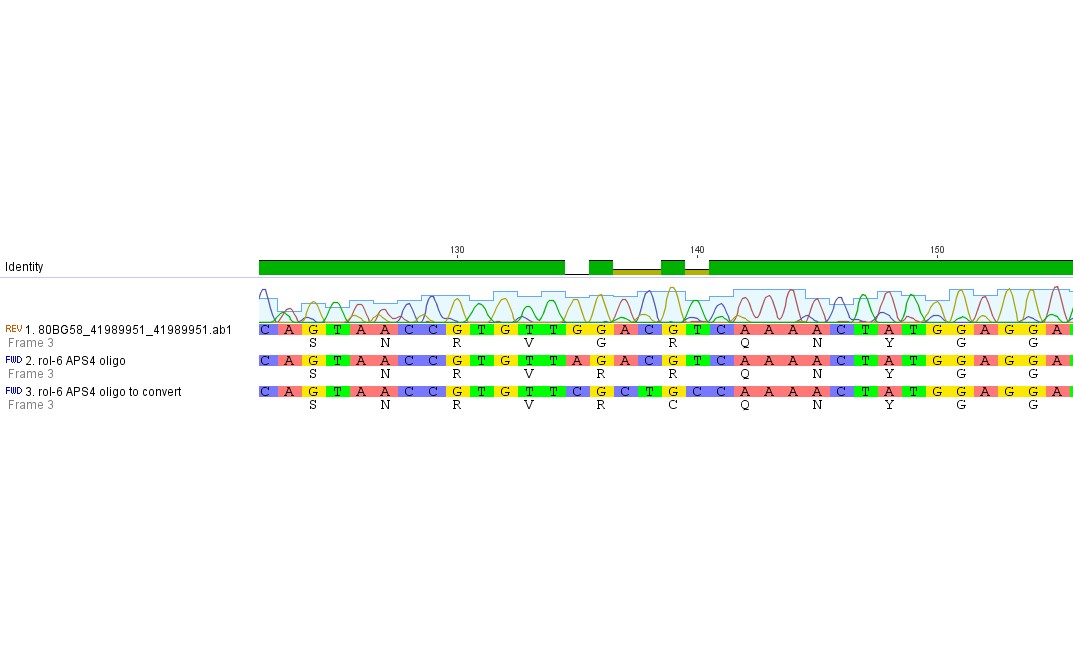


*Arh-rol-6.1* Line 5

*Arh-rol-6.1* wild type

ssDNA conversion

fragment

70

71

69

68

72

73

74

Figure S7

A single point mutation in *Arh-rol-6.1* line 5 results in an Arginine (R) to Glycine (G) amino acid substitution at position 70 in the predicted amino acid sequence (changing motif RVRR to RVGR).


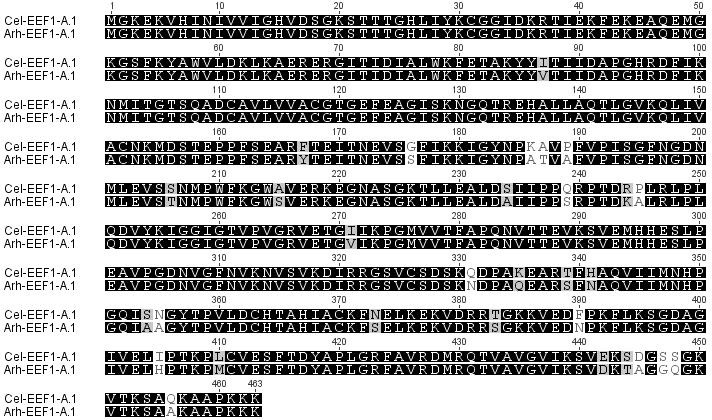


Figure S8

Full length alignment of predicted EEF-1-A.1 proteins encoded by *Cel-eef1-A.1* (P53013) and *Arh-eef1-A.1* (MH124553). The sequences exhibit 94% identity.

ATGACTGCTCCAAAGAAGAAGCGTAAGGTACATCATGCCAAATCAAAACTAACGTATACATAGCAACATGAGGGCCTTGTCTAGAATTAGCTTTCAATGCTGTAGCAGATAAGGAAAATACTGCATTTTCTGTAACCGAAATATCGCGAATATCACTTTCGGCTGCAGCAAATGAGGTATACTTCTCAAAGCCATTTTCAGCATAAAATGAAGTTACTCGGCCCTGAGATTCTAATGTAATCCCGTCTTCAACACAGAAAACTTACGTTAATATTCCCGCACCAAAGCAACTCCTCAAAAGGATCAAAAGATAGCGAAGTAATTGGTAAACCTGATAAATTCGACTCAATCCTTGAGAGGTATCCAAAATTACCAATACCAGGTGTCCACCAAAAAATCAAAATCAACAAAGTTTTACTCAGAAACGAACCTCAACTTCTTCATTTTCTACATCTTCTATTGTATTCTCCATAATGCTTAATCATTTAACGATATTTTGTTTCCGAACAAAAAATTTATCACAAAGTCATTCGACTAAAAGCATCGTTAAAAATATCCACCAAAGGAGAAAGCTTCAATAACCAATTATCAAAAATTAGAACCCTGTGATTAAATGTCAAAAAATCGATTTAAATTTCAAGACACAATTTCCAAAGAACTGTTCATTTTATCGATTTTTCCCGTGAAGATGTATGGTTCGCGCCATTCAATGTTTGAATTACGGTAGAAATCGTGGCAAGATATGTAGCTTAGCCGCGCCAGCAATTTTACGAAATTTTAGGCCCATAAGACTTTCAGCACCAGTTGTTTTCAGTCGTTCATTCCATAGTTACATCTACTTGTTTGTAGCAATCTTGTATTCTTTTAATTTCTTTTGTTCTTAATTATATTTAAAACGTTTTAAAAGAAGTTTAAATTTTTATTATAGTTGGGAAAAACTTTGCTCACTCCACAGCCCGGTAGAAAAAATGAGCGAGCTGATCAAGGAGAACATGCAC

Figure S9

Nucleotide sequence of the gBlock gene fragment (IDT) used to insert the promoter of *Arh-eef-1-A.1* upstream of the TurboRFP gene in the *Ppa*::TurboRFP vector ^2^. The predicted promoter region of *Arh-eef-1-A.1* is shown in black and corresponds to a 926 bp region immediately upstream of the predicted ATG transcriptional start codon. Sequence corresponding to short regions of the *Ppa*::TurboRFP vector flanking the unique *KpnI* restriction site, incorporated to allow seamless insertion of the promoter region by Gibson cloning (NEB), are highlighted in red.


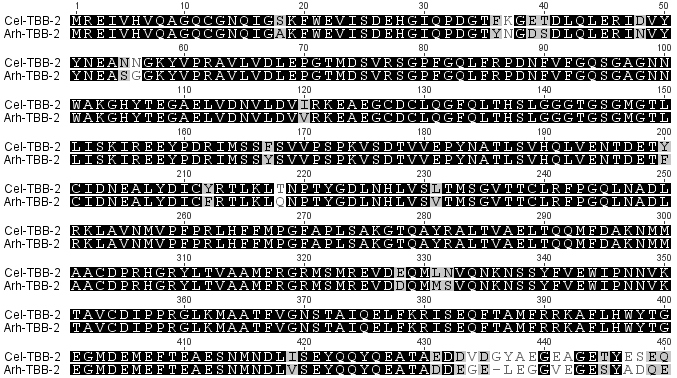


Figure S10

Full length alignment of predicted TBB-2 proteins encoded by *Cel-tbb-2* (P52275) and *Arh-tbb-2* (MH124554). The sequences exhibit 93% identity.

TAGCAAACTGGGGCACAGATGATCCGAGCTCATCTCCGCATATGCACAATCGACAATAAATTATGTTGCTTCCTGATCTATGCTTACCTTAATGTTTTATTTATTGCAATGTTATCTAAATGAACTGGCTTAACCAAAGCACTTGTTTATGCATTCTTACACAACAATACTGCTATCGGCACGCACTTTCACAACACAACGAGTGGGCTTAATATGACGAATAATCAATCATGGTTTCTGTCTGATCAACGAAATAAAATGTTAATGGTAATTCACTGGCCGTCGTTTTACAACGTCGTGACT

Figure S11

Nucleotide sequence of the gBlock gene fragment (IDT) synthesised to replace the *P. pacificus* *rpl-23* 3’ UTR with the predicted *Arh-tbb-2* 3’ UTR, downstream of the TurboRFP gene, in the *Ppa*::TurboRFP vector ^2^.The nucleotide sequence of the predicted 3’ UTR of *Arh-tbb-2* is shown in black and possible polyadenylation and cleavage sites (AAUAAA) are underlined*.* DNA sequences corresponding to sections of the *Ppa*::TurboRFP vector to allow insertion by Gibson cloning (NEB) are highlighted in red.


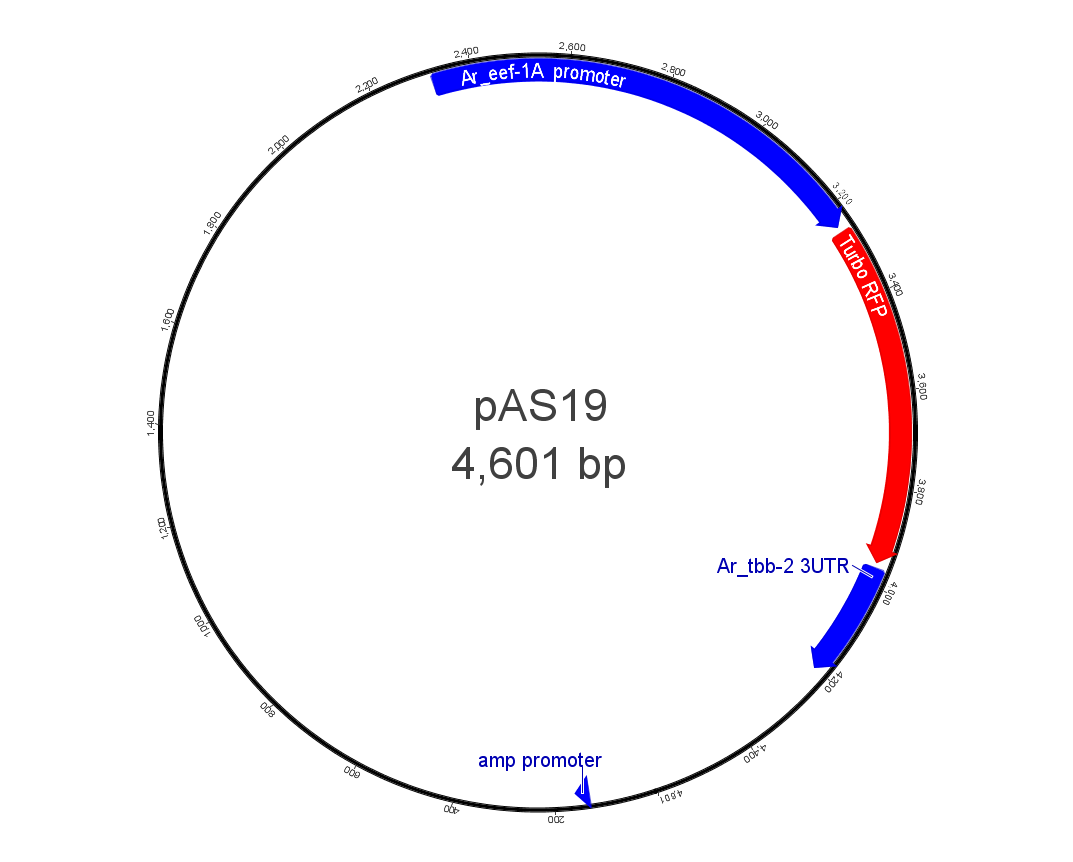


Figure S12

Schematic representation of the pAS19 *A. rhodensis* expression vector.

**References**

1 Kramer, J. M. & Johnson, J. J. Analysis of mutations in the *sqt-1* and *rol-6* collagen genes of *Caenorhabditis elegans*. *Genetics* **135**, 1035-1045 (1993).

2 Schlager, B., Wang, X., Braach, G. & Sommer, R. J. Molecular cloning of a dominant roller mutant and establishment of DNA-mediated transformation in the nematode *Pristionchus pacificus*. *Genesis* **47**, 300-304 (2009).
